# Potential Application of Ovalbumin Gel Nanoparticles Loaded with Carvacrol in the Preservation of Fresh Pork

**DOI:** 10.3390/gels9120941

**Published:** 2023-11-30

**Authors:** Ruyi Zhang, Guangwei Xu, Yujie Su, Shengqi Rao

**Affiliations:** 1 State Key Laboratory of Food Science and Technology, Jiangnan University, Wuxi 214122, China; 7230112055@stu.jiangnan.edu.cn (R.Z.); suyujie@jiangnan.edu.cn (Y.S.); 2 College of Food Science and Engineering, Yangzhou University, Yangzhou 225127, China; 15705271752@163.com

**Keywords:** ovalbumin, gel nanoparticles, carvacrol, fresh pork, preservation

## Abstract

Plant essential oil has attracted much attention in delaying pork spoilage due to its safety, but its low antibacterial efficiency needs to be solved by encapsulation. Our previous research had fabricated a type of ovalbumin gel nanoparticles loaded with carvacrol (OCG*_n_*-2) using the gel-embedding method, which had a high encapsulation rate and antibacterial activity. The main purpose of this study was to further evaluate the stability and slow-release characteristics of OCG*_n_*-2 and potential quality effects of the nanoparticles on the preservation of fresh pork pieces during 4 °C storage. The particle test showed that the nanoparticles had better heat stability below 85 °C and salt stability below 90 mM. The in vitro release study indicated that the carvacrol in OCG*_n_*-2 followed a Fickian release mechanism. The pork preservation test suggested that the OCG*_n_*-2 coating treatments could remarkably restrict the quality decay of pork slices compared to free carvacrol or a physical mixture of ovalbumin and carvacrol treatment. Nano-encapsulation of ovalbumin is beneficial to the sustained release, enhanced oxidation resistance, and improved antibacterial activity of carvacrol. The study suggested that ovalbumin gel nanoparticles embedded with carvacrol could be applied as an efficient bacterial active packaging to extend the storage life of pork.

## 1. Introduction

Among the common meat products, pork is consumed in huge quantities throughout the world. However, a mass of polyunsaturated fatty acids in pork is harmful because biochemical reactions such as oxidation and microbial spoilage can bring about color changes and rancid taste [1]. The deterioration of meat during manufacturing and storage involves three mechanisms: microbial spoilage, lipid oxidation, and enzymatic autolysis. Red meat such as pork [2], beef [3], and mutton [4] are frequently related to outbursts mainly because of the existence of *Salmonella* spp., *Staphylococcus* spp., *Clostridium* spp., and *Listeria* spp. In recent years, *Salmonella* has been reported as the main reason for foodborne disease transmission leading to hospitalization [5]. *Salmonella* in the feces can contaminate the surface of the carcass during the slaughter process, and intestinal diseases and bacterial infections can probably be generated after accidental consumption [6]. Therefore, contamination with foodborne human pathogens, especially by *Salmonella*, is becoming an increasing concern for the pork industry. The spoilage factors in meat products sometimes represent a high risk for producers’ interest and poses a threat to consumer health. Therefore, preventing the impact that the storage process has on *Salmonella* prevalence is a critical step in developing strategies for pathogen reduction in meat and can guarantee economy and safety of the meat processing industry.

In meat preservation, it is common that the addition of different preservatives in various meat products could prevent lipid oxidation, delay rancidity, and improve color sharpness. Despite the excellent efficiency and stability of synthetic preservatives, in the past several years, researchers have taken increasing interest in replacing them with natural preservatives such as plant essential oil (PEO) on account of their potential impacts on toxicity, carcinogenesis, teratogenicity, and mutagenicity [7]. PEO, frequently used by meat producers, is extracted from specific plants, such as clove, thyme, and ginger [8,9,10]. Many of them are generally recognized as safe (GRAS) for use in the food industry, as declared by the United States Food and Drug Administration (FDA) [11]. Carvacrol (Car), purified from the secondary metabolites of oregano and thyme, has strong abilities against both microorganisms and oxidization [12]. Nonetheless, the application of Car in food processing is blocked because it is quickly volatile and hardly soluble in water.

Encapsulation, regarded to be a burgeoning and current method, can capture and improve the solubility of PEO, and likewise effectively prevent sustained release during processing and action, to manufacture a nano-delivery system in the form of nanoparticles [13]. The use of active food packaging, which utilizes PEO-loaded nanoparticles as active agents, has become gradually attractive due to their properties against pathogenic microorganisms and oxidation as well as stability by resisting varieties of environmental impacts [7]. Evidently, gel nanoparticles represent intricate, three-dimensional nanoscale biomacromolecular constructs that are intricately cross-linked, capable of accommodating substantial fluid volumes, and, consequently, a noteworthy quantity of active constituents [14]. Innovative hydrogel carriers, varying in matrix sizes from micrometers to nanometers, bestow heightened transport capabilities. Gel nanoparticles seamlessly amalgamate the advantages of hydrogels and nanoparticles, boasting traits such as water solubility, distinctive adhesive properties, a spacious specific surface area amenable to manipulation, an elevated loading capacity, meticulously regulated delayed release kinetics, and a superior degree of biocompatibility and biodegradability [15]. Protein (e.g., whey protein [16], gliadin [17], zein [18], and ovalbumin) is one of the most generally employed materials for the manufacture of esculent nanoparticles since it can be assembled by covalent or noncovalent bonds. Ovalbumin (OVA), the most plentiful protein component in egg white protein (EWP), comprises about 192 hydrophobic amino acids and about 128 of which are electropositive/electronegative; these demonstrate the feasibility of OVA as an efficient carrier for agent delivery [19].

Our previous studies have explored the physicochemical properties and antibacterial properties of ovalbumin–carvacrol (OVA-Car) nanocomposites prepared by the oil-in-water method [20] and gel-embedding method [21]. It was found that OVA-Car prepared by gel-embedding method had better properties than those of OVA-Car from the oil-in-water method. Moreover, among different pH conditions (2, 5, 7, and 9), which can affect gel microstructure, OCG*_n_*-2 (OVA gel nanoparticles prepared loaded with Car at pH 2) was selected because its gel structure was more dense and stable, and has higher encapsulation efficiency contributing to higher sterilization rate.

However, previous studies have only explored the physicochemical properties and antibacterial properties of nanoparticles while application tests on preserving fresh pork are lacking. Hence, the purpose of this study was to apply OCG*_n_*-2 to evaluate its influence on the quality characteristic of fresh pork contaminated with *Salmonella* during 4 °C storage. This is based on the investigation of the influence of environmental stresses on the stability of nanoparticles and the release profiles of Car from OCG*_n_*-2, which have the possibility to be exploited as an effective economic approach for meat preservation. In the long term, this study may offer new creative approach into the development of delivery systems with improved biological preservatives.

## 2. Results and Discussion

### 2.1. Form Analysis

**Figure 1:**
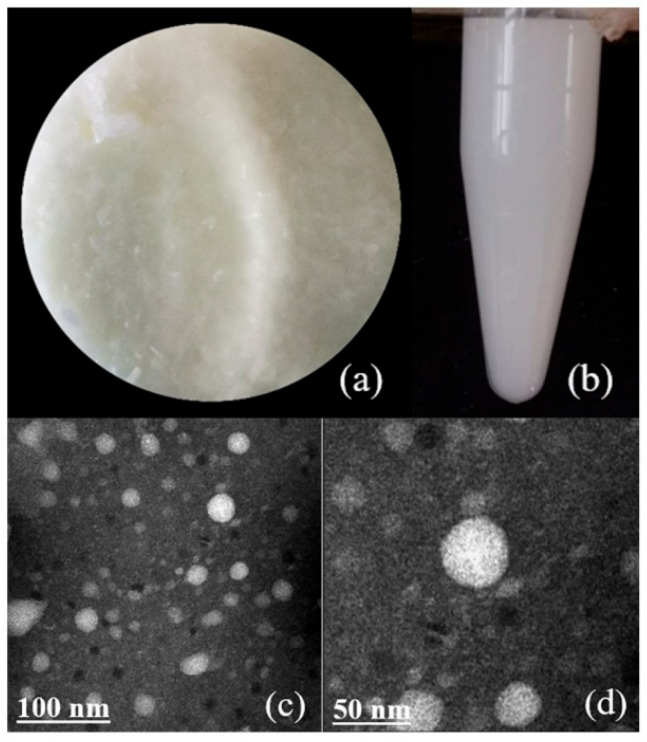
(**a**,**b**) Sample morphology of OCG*_n_*-2. (**c**,**d**) Transmission electron microscope images of OCG*_n_*-2.

The solid sample was a light-yellow powder with tiny and uniform particles (Figure 1a). It was easily soluble in water and appeared as a white liquid with high turbidity at high concentrations (Figure 1b). The nanoparticle image demonstrated spherical and regularly distributed particles, which had good homogeneity and stability without adverse reactions during the preparation process (Figure 1c,d). The results of TEM indicated that the size distribution of most nanoparticles was between 40 and 60 nm. The reason for the larger particle diameter might be that the protein layer outside the single particle swells and/or the single particle agglomerates when dispersed in the solution. OVA encapsulation is indicated to improve the delivery mode of antibacterial agents on pathogenic bacteria and provided a reference for future research.

### 2.2. Effects of Environmental Stresses on the Stability of OCG_n_-2

**Figure 2:**
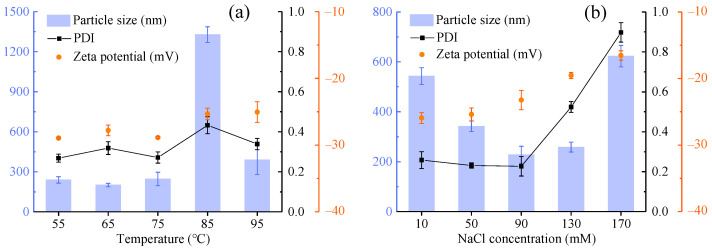
Effect of environmental factors of temperature (**a**) and NaCl concentration (**b**) on the particle size, polydispersity index (PDI) and zeta potential of OCG*_n_*-2. Values are means ± standard deviations, indicated by error bars.

Figure 2a depicted the impact of thermal treatment in the temperature range of 55–95 °C for 15 min on the particle size, PDI and zeta potential of the solutions. Noticeable trends included an increase in particle size and a widening of the size distribution at 85 °C on account of the poor thermal stability of OCG*_n_*-2. At other temperatures, their average particle size remained below 350 nm, while the PDI fluctuated slightly around 0.3 because the composite particles tend to aggregate, resulting in larger particle sizes than expected. When the temperature fell below 85 °C, the zeta potential exhibited slight fluctuations between −27 mV and −29 mV. This indicated that OCG*_n_*-2 successfully withstood thermally induced aggregation at this stage. However, surpassing 85 °C diminished the net charge content, leading to particle aggregation and consequently an upsurge in the measured particle size. Together, the above results indicated that OCG*_n_*-2 became stable under low-temperature treatment. 

As shown in Figure 2b, the effect of NaCl concentration on the stability of OCG*_n_*-2 was assessed by assessing the particle size, PDI and zeta potential of the solution at different concentrations of NaCl (10–170 mM). The particle size of OCG*_n_*-2 experienced a slight decrease within the range of 10 to 90 mM NaCl. This phenomenon can be attributed to the capacity of low NaCl concentrations to induce protein-based nanoparticle structures to contract, leading to a smaller particle size. A comparable trend was observed in composite condensed nanoparticles crosslinked with tannic acid [22]. Starting with the introduction of a 90 mM NaCl solution, the PDI of the solution exhibited a notable and rapid increase in response to an elevated ionic strength, surpassing the changes observed at the 0.3 level. Ultimately, the nanoparticles displayed the greatest particle size and PDI in 170 mM NaCl solution. The net charge of OCG*_n_*-2 exhibited a gradual decline as the NaCl concentration increased. Particularly noteworthy is that the net charge dropped below 20 mV upon surpassing 130 mM, signifying significant particle aggregation. These observations suggest that the electrostatic repulsion and steric hindrance between nanoparticles have been severely compromised due to the screening effects induced by high ionic strength [23]. As a result, instability ensues, leading to extensive nanoparticle aggregation. A similar phenomenon was also observed in the NaCl stability results of *Camellia* seed albumin nanoparticles [24]. In summary, OCG*_n_*-2 subjected to temperatures below 85 °C or treated with NaCl concentrations below 130 mM retained a uniform small particle size. This might make the contact area between the samples and pathogenic microorganisms large enough to facilitate the diffusion of the antibacterial active ingredients of OCG*_n_*-2 on the bacterial cell membrane.

### 2.3. In Vitro Release Studies

**Figure 3:**
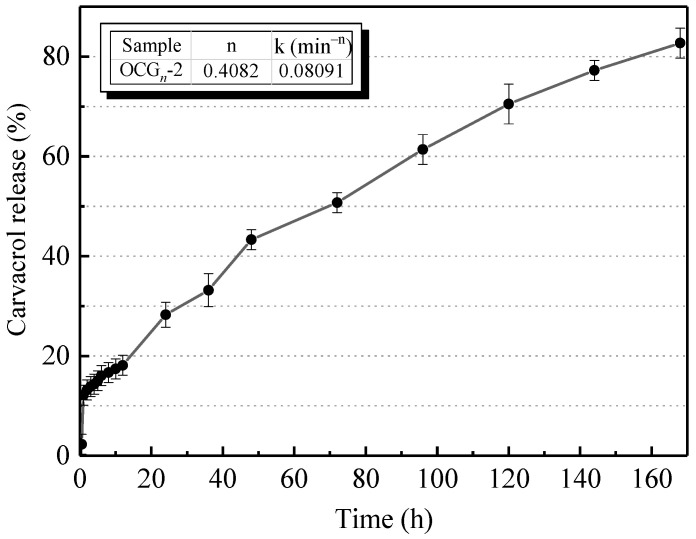
The *n* and k values for the Car release and in vitro release profiles of Car from OCG*_n_*-2 nanoparticles. Values are means ± standard deviations, indicated by error bars.

The in vitro release profiles of Car from the nanoparticles under ambient temperature are shown in Figure 3. The results showed that the release of Car from OCG*_n_*-2 was divided into two stages according to the rate of release. A burst release of Car was discovered during the preliminary 60 h of the initial phase. Car was released up to 12.13% in the buffer solution. The release at this stage was mainly due to the free Car attached to the surface of the nanoparticles [25]. The release speed of the second stage drops slightly, that is, between 60 h and 168 h, when the accumulated amount of Car released was 82.71% in the buffer solution.

For the spherical nanoparticles in this study, *n* = 0.4082, which is less than 0.45, indicating that the Fickian release mechanism was followed [26]. The linear relation of ln ($\frac{M_{t}}{M_{0}}$) versus ln(t) was used to measure the value of *n* and k.

### 2.4. Meat Storage Test

#### 2.4.1. pH Test

**Figure 4:**
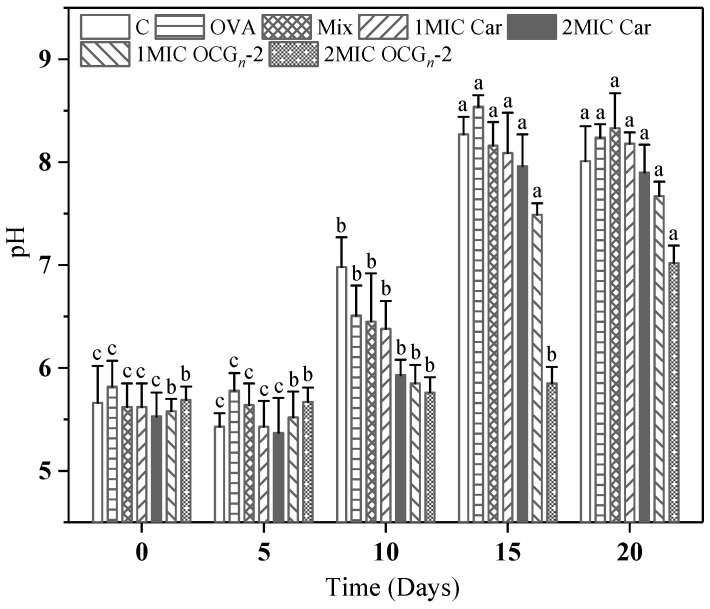
Changes in pH value of pork samples with different treatments during 4 °C storage. Values are means ± standard deviations, indicated by error bars. Values with different letters of each sample indicate significant difference at *p* < 0.05.

The pH level of fresh, unprocessed pork typically falls within the range of 5.18 to 6.17 [27]. As exhibited in Figure 4, the initial pH was 5.53–5.82 at 4 °C. At the beginning of the storage, we observed a slight decline in pH value, attributing to the proliferation of lactic acid in pork samples [28]. The pH reached the upper limit for fresh meat (pH = 6.8) in around 10 days. The pH rose sharply especially after the 15-day storage period, ranging from 7.96 to 8.54 except for two meat samples coated with OCG*_n_*-2. This could be ascribed to the accretion of ammonia and the dissolution of the amino acids for the reason that bacteria used and consumed amino acids [29]. It could be noticed that the spoilage degree of pork samples coated with OCG*_n_*-2 was less severe than free Car groups, indicating the ovalbumin nano-encapsulation enhanced the rot-fastness ability of Car. The pH of the meat sample treated with 2 MIC OCG*_n_*-2 did not change significantly in the first 15 days, indicating that 2 MIC OCG*_n_*-2 could restrain the chemical changes in the meat sample, accordingly achieving the purpose of preservation.

#### 2.4.2. Weight Loss Test

**Figure 5:**
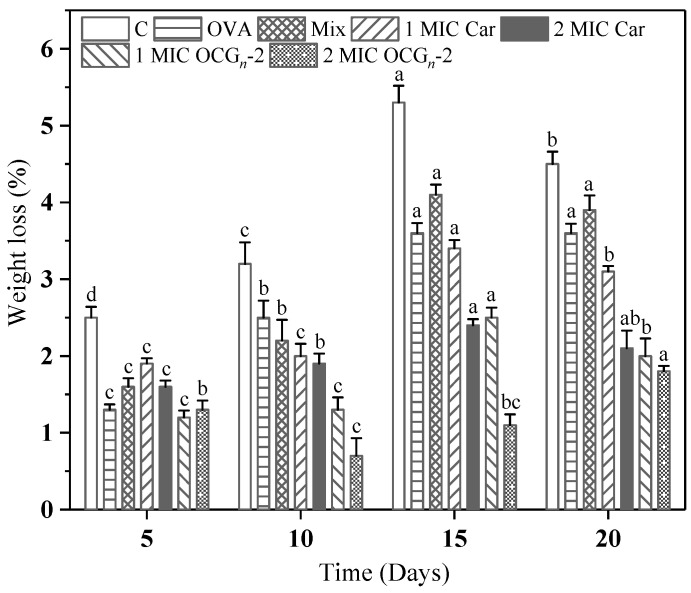
Changes in weight loss of pork samples with different treatments during 4 °C storage. Values are means ± standard deviations, indicated by error bars. Values with different letters of each sample indicate significant difference at *p* < 0.05.

Water loss can result in the reduction of meat weight, primarily attributable to the irreversible transformation of the protein colloid, thus preventing the moisture in the gel structure from being retained and flowing out of the tissue. As shown in Figure 5, the untreated group exhibited a notably greater decline in weight compared to the other treatment groups, and this trend intensified with the passage of time during storage. It was because, during the storage process of pork, the water-holding capacity of pork became worse because of microbial contamination, which exacerbated the water loss. According to the general trend, the degree of the weight loss of pork peaked in the initial 15-day storage period and then decreased slightly at 20 days. It was speculated that the free water in the pork samples had almost completely evaporated during the 15-day storage period of refrigeration, leaving the non-volatile bound water. It could be seen that the weight loss of samples coated with OCG*_n_*-2 after 10 days was remarkably lower than that of other treatment groups. In contrast to the free Car group, the weight loss of pork samples coated with 2 MIC OCG*_n_*-2 was reduced by up to 43%. The findings indicated that nanoparticles were proficient in significantly mitigating the rate of weight loss in pork, indicating that ovalbumin nano-encapsulation improved the action efficiency of free Car.

#### 2.4.3. Color Measurement

**Figure 6:**
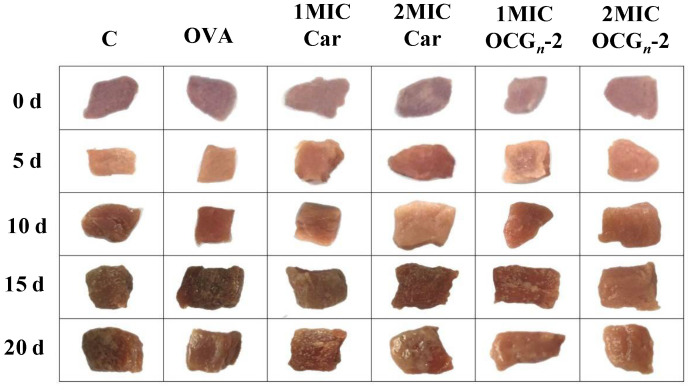
The appearance of different pork samples stored at 4 °C for 20 days.

The color change of the pork surface is an important indicator that reflects its visual quality, which influences consumer acceptability. L*, a*, and b* of the selected color parameters are generally used to estimate the visible quality of fresh meat [30]. The color parameters of different pork samples stored at 4 °C are exhibited in Table 1. With the extension of time, L* and b* values showed an upward trend, while the a* value showed a downward trend. On the micro level, as meat deteriorated, deoxymyoglobin (purple) or oxymyoglobin (red) was oxidized to a brown color [31]. L* values were barely influenced by an oxidation reaction, just revealing a slight growth with the storage period. The increase in protein oxidation over the storage period resulted in reduced water retention and hence greater dispersion of light [32]. The red color (a*) of meat is often regarded as an indicator of meat freshness [33]. At 4 °C, the initial range of the a* value in the study spanned from 10.81 to 15.23. These distinctions among treatments might be due to the dosage of reagents used that might impart color to the samples. As depicted in Figure 6, the visual aspects of the various pork samples stored at 4 °C substantiate the dependability of the parameters.

#### 2.4.4. Lipid Oxidation

**Figure 7:**
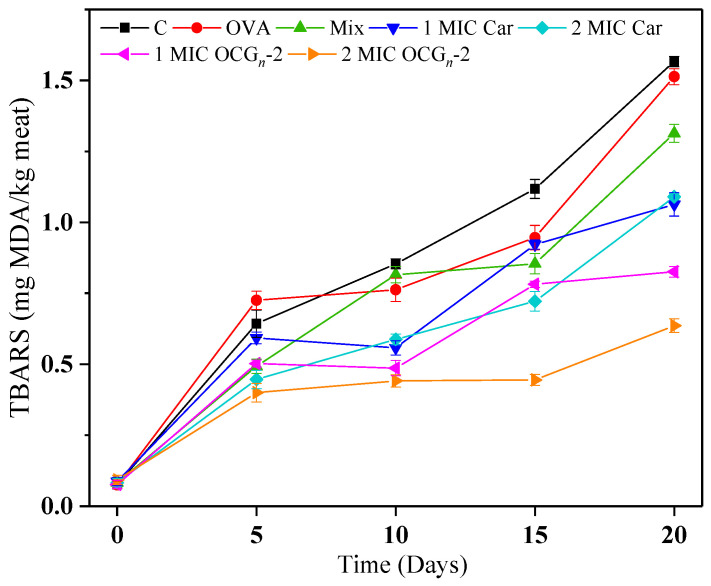
Changes in lipid oxidation of pork samples with different treatments during 4 °C storage. Values are means ± standard deviations, indicated by error bars.

Lipid oxidation stands as the predominant factor contributing to the deterioration in meat quality due to the occurrence of rancidity, smelly substance, and other adverse reactions, leading to the decrease in shelf life and public acceptability [34]. The assessment of lipid oxidation in the pork samples was carried out through the measurement of the TBARS value. A higher TBARS value indicates a greater degree of lipid oxidation in the pork samples. As depicted in Figure 7, during the initial phase of storage at 4 °C, TBARS values exhibited fluctuations within the range of 0.075 to 0.093 mg MDA/kg. As anticipated, TBARS values in all seven treatments exhibited a significant increase (*p* < 0.05) over the storage duration, signifying the advancement of lipid oxidation. It was observed that the TBARS value of the free Car group showed severe lipid oxidation than samples coated with OCG*_n_*-2, indicating that the ovalbumin nano-encapsulation enhanced the exertion of inoxidizability of the free Car. Zhang [35] proposed that in contrast to free essential oils, nano-encapsulated essential oils demonstrate heightened antimicrobial and antioxidant potency. In addition, during a 15-day storage period, the TBARS values of 2 MIC OCG*_n_*-2-treated groups consistently remained below 0.5 mg MDA/kg, a threshold considered sensorially acceptable to prevent rancidity [36].

#### 2.4.5. Protein Oxidation

**Figure 8:**
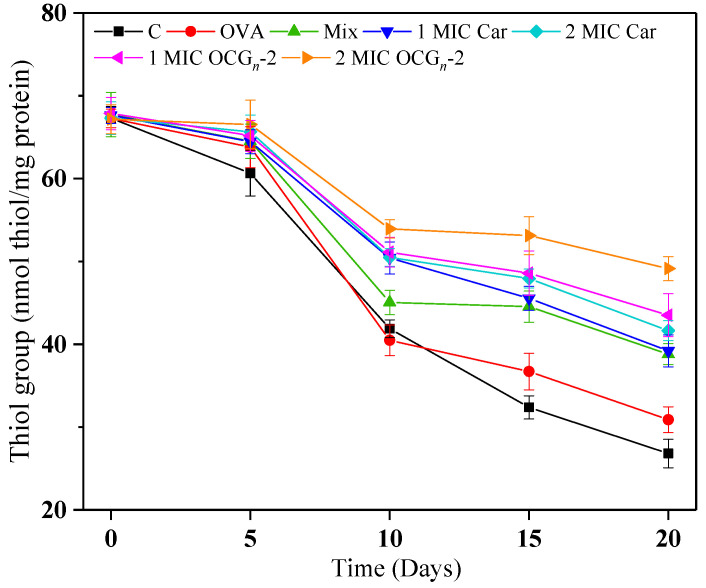
Changes in protein oxidation of pork samples with different treatments during 4 °C storage. Values are means ± standard deviations, indicated by error bars.

The presence of thiol groups resulting from protein oxidation within the samples is depicted in Figure 8. Within a dose effect, OCG*_n_*-2 with added 2 MIC indicated the furthest restraint effect on the protein oxidation; the thiol contents decreased by 26.8%, lower than that of the free Car group. It could be judged that ovalbumin nano-encapsulation conduced free Car to decrease the loss of thiol contents. As described by Xiong [37], the controlled release mechanism of the nano-encapsulation system demonstrated the ability to enhance the antioxidant efficacy of essential oils in meat products. Additionally, it is common knowledge that phenolics are characterized by hydroxyl groups connected to the aromatic ring, which can provide electrons that can neutralize free-radical reactions, resulting in the inhibition of oxidizing reaction [38].

#### 2.4.6. Microbiological Analysis

**Figure 9:**
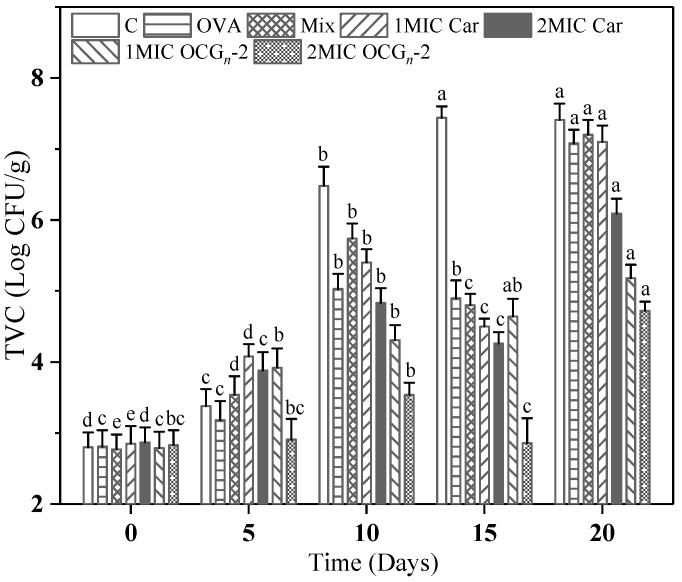
Changes in total viable counts (TVC) of pork samples with different treatments during 4 °C storage. Values are means ± standard deviations, indicated by error bars. Values with different letters in each row indicate significances different at *p* < 0.05.

The change in TVCs of pork samples is revealed in Figure 9. The initial TVC exhibited a relatively narrow range, spanning from 2.87 to 4.12 log CFU/g. Over the 4 °C storage, the pork samples coated with 2MIC OCG*_n_*-2 had a TVC remarkably lower than that of the control samples. According to Huang et al. [39], a TVC value of 7 log CFU/g can be established as the threshold indicative of the freshness and quality of pork. The pork samples treated with OCG*_n_*-2 were far below this threshold value compared with free Car groups after 20 d during 4 °C storage, demonstrating that the fabricated nano-delivery system could effectively help free essential oil to persistently protect the pork from spoilage microorganisms within a certain edible period.

## 3. Conclusions

The present study investigated the application of OCG*_n_*-2 developed previously for fresh pork preservation. Under certain conditions (thermal treatment and ionic strength), OCG*_n_*-2 could remain relatively stable, while different durations greatly affected the controlled-release process of Car. Application of OCG*_n_*-2 reduced discoloration of fresh pork, successfully decreased lipid and protein oxidation, prevented weight loss and pH change, and inhibited microbial growth. Compared with free Car, OCG*_n_*-2 with a concentration of 0.32 mg/mL (i.e., 2 MIC) provided extended shelf life of fresh pork. To sum up, the results demonstrated broad application prospects of OCG*_n_*-2 in pork preservation processing in comparison with free essential oil. Nevertheless, it is necessary to further uncover the underlying mechanism and to evaluate the possible physical toxicity.

## 4. Materials and Methods

### 4.1. Materials and Chemicals

Fresh eggs were obtained from a local supermarket (Yangzhou, China). Car (99%) and 1,1,3,3-tetraethoxypropane were obtained from Sigma-Aldrich (St. Louis, MO, USA). The 5,5′-dithiobis-(2-nitrobenzoic acid) was purchased from Biological Technology Co., Ltd. (Shanghai, China). All other chemicals were of analytical grade. Fresh pork loins were obtained from a local market (Yangzhou, China).

### 4.2. Preparation of OCG_n_-2 Nanoparticles

The preparation method was referred to our previous study [21]. An equal volume of deionized water was mixed with the separated egg white, and then the pH was adjusted to 2.0 with HCl (1 M). After undergoing magnetic stirring for a duration of 3 h, the suspension was subsequently subjected to centrifugation at 8000× *g* for a period of 15 min. Egg white protein supernatant was collected, and 31 mg/mL Car was added. The mixture was agitated at 25 °C for a span of 3 h within a water bath maintained at 90 °C for 30 min, after which it was promptly cooled to 4 °C by immersing it in an ice bath. OVA-Car gel at pH 2 (OCG-2) formed after refrigeration overnight at 4 °C. The gels were disintegrated through stirring and homogenized with the aid of an ultrasonic processor to achieve uniform OVA-Car gel nanoparticles at a pH level of 2 (OCG*_n_*-2).

### 4.3. Effect of Environmental Stress on the Stability of OCG_n_-2

#### 4.3.1. Effect of Thermal Treatment

The samples were dissolved in an equal volume of phosphate buffer at a pH of 2. The sample solutions were heated at different temperatures (55, 65, 75, 85, and 95 °C) for 15 min. The treated samples were then cooled in a water bath and diluted to 0.5 mg/mL to scale the physical stability. In addition, the particle size, analyzed through the procedure in Section 4.5, was used to denote the stability of the solution [40].

#### 4.3.2. Effect of Ionic Strength

The samples were dissolved in an equal volume of phosphate buffer at a pH of 2. The sample solutions were diluted with different concentrations (10, 50, 90, 130, and 170 mmol/L) of NaCl solutions and stored (4 °C). The particle size, analyzed by the procedure in Section 4.5, was used to express the stability of the solution.

### 4.4. Transmission Electron Microscopy (TEM) Analysis

The morphology analysis of nanoparticles by TEM was conducted in OCG*_n_*-2 with a Tecnai 12 (Philips, Amsterdam, The Netherlands) operated at 100 kV. The sample previously diluted in ultrapure water was placed on carbon-coated copper grids and then observed under the microscope after drying.

### 4.5. Particle Size and Polydispersity Index (PDI) Determination

Dynamic light scattering was employed for the determination of particle size distribution utilizing the Zetasizer Nano Es90 instrument (Malvern Instruments, Worcestershire, UK). Approximately 1 mL of the test samples was placed in a Marvin measuring chamber, whose temperature was set to 25 °C.

### 4.6. In Vitro Release Studies

A certain amount of OCG*_n_*-2 was incubated in a test tube containing 3 mL of pH 2 phosphate buffer at 25 °C, and samples were taken for centrifugation at specific time points. The total cumulative amount of Car released was measured by a UV–Vis spectrophotometer scan (UV-2550, Shimadzu, Kyoto, Japan) at 275 nm, and the volume of the released medium (mL) was converted into the released amount (g) [41].

The mechanism of Car release was studied using Equation (1):

(1) $$\frac{M_{t}}{M_{0}}={\mathrm{kt}}^{n}$$

where *M*_t_ is the content of Car released in a certain period, *M*_0_ is the content of Car encapsulated in the delivery system, t represents the release duration, k denotes the kinetic constant governing the system, and *n* is a diffusional exponent used to determine the release mechanism.

### 4.7. Application of Nanoparticles on Fresh Pork

#### 4.7.1. Preparation of Fresh Pork Samples

The preparation of pork samples adhered to the methodology outlined by Wang et al. [9], albeit with certain tailored adjustments. Upon the removal of discernible fat and connective tissues, the pork loins were meticulously sectioned into diminutive segments of 2 cm × 2 cm × 2 cm (about 10 ± 1 g each). A total of 105 pork samples were randomly chosen and apportioned among seven distinct treatment cohorts. The formulation and final effective concentration of prepared coating solutions used in each group were listed in Table 2. 

The pork samples were fully submerged in the meticulously prepared coating solutions for a 30 s. Subsequently, any surplus solution adhering to the surface of the pork samples was eliminated through a 10 min air-drying process on the countertop. After that, pork samples were, respectively, placed in the disposable sterile polystyrene tray and packed into individual polyethylene self-seal bags (10 cm × 15 cm), and then stored at 4 °C. The evaluation encompassed measurements of pH levels, weight loss, lipid oxidation, protein oxidation, and the overall microbial count on days 0, 5, 10, 15, and 20.

#### 4.7.2. pH Measurement

After the surface liquid was removed, the pork samples were homogenized and centrifuged. The specimens were measured by an easy pH meter (PHS-3C, Shanghai, China). The change in pH value of differently treated samples was measured at a specific time interval (every 5 day) [42].

#### 4.7.3. Weight Loss Test

To evaluate the availability of the nanoparticles in reducing myoserum loss, a weighing method was used. Liquid on the pork samples surface was eliminated before measurement. The weight loss of the pork samples can be calculated using Formula (2) as follows:

(2) $$Weight\;loss\;(\%)=\frac{m_{0}-m_{n}}{m_{0}}\times 100\%$$

where *m*_0_ is the mass of the untreated fresh pork sample, and *m_n_* is the mass of the pork sample on day *n* during the storage period.

#### 4.7.4. Color Measurement

The colors of the pork samples were determined by a color sensor (SC-80C Automatic Color Sensor; FTC, Stirling, VA, USA), following the method of [43]. The values were determined based on color parameters, including L* (representing lightness), a* (indicating redness), and b* (signifying yellowness).

#### 4.7.5. Lipid Oxidation

Thiobarbituric acid reactive substances (TBARS) assay was used to determine the lipid oxidation of pork samples, designed by [44] with some modification. In brief, 10 g of the pork samples were blended with 20 mL of trichloroacetic acid (TCA, 7.5%, *w*/*v*) for 1 h, after which malonaldehyde (MDA) was extracted. A volume of five milliliters of the clarified supernatant was combined with an equivalent volume of 0.02 M 2-thiobarbituric acid (TBA) and subjected to a 30 min incubation within a vigorously boiling water bath. Subsequent to cooling in an ice-water bath, the absorbance of the samples was quantified at 530 nm using a UV–Vis spectrophotometer scan (UV-2550, Shimadzu, Japan). A standard curve for 1,1,3,3-tetraethoxypropane was established to ascertain the MDA content. The outcomes were presented in milligrams of MDA (equivalent) per kilogram of the sample.

#### 4.7.6. Protein Oxidation

The assessment of protein oxidation was carried out employing the 5,5′-dithiobis-(2-nitrobenzoic acid) (DTNB) method, raised by Ellman (1959) and modified by [45]. In brief, the pork sample (2 g) was homogenized in 25 mL of 5% (*w*/*v*) sodium dodecylsulfate (SDS) in 0.1 M phosphate-buffered saline (pH 8.0), and then 0.5 mL filtered aliquot was mixed with 0.5 mL 10 mM DTNB in 0.1 M Tris buffer (pH 8.0). Following light-protected incubation at 25 °C for a duration of 30 min, the sample absorbance was quantified at 412 nm using a spectrophotometer, and the results were converted into the expression of nanomoles of thiol per milligram of protein.

#### 4.7.7. Microbiological Analysis

The samples from different treatment groups (each 1 g) were completely homogenized with sterile normal saline (each 9 mL) and serially diluted. The total viable count (TVC) was ascertained through the plate counting technique, involving an incubation at 37 °C for a duration of 48 h. The data were presented as the logarithm of colony-forming units per gram (log cfu/g).

#### 4.7.8. Statistical Analysis

Each experiment was performed in triplicate, and the results were provided as the mean ± standard deviation. In order to determine the significance of differences between the means, a thorough statistical analysis was conducted using SPSS 19.0 software. Statistical significance was deemed to be achieved at a threshold of *p* < 0.05.

## Figures and Tables

**Table 1:** Changes in color characteristics of pork samples stored at 4 °C for 20 days. Values are mean ± standard deviation. Values with different letters in each row indicate significant difference at *p* < 0.05.

Attributes	Treatment Groups	Storage Days				
		0	5	10	15	20
L*	C	36.38 ± 1.31 ^b^	35.29 ± 1.87 ^b^	36.74 ± 0.94 ^b^	39.44 ± 1.61 ^b^	46.53 ± 0.92 ^a^
	OVA	40.00 ± 1.47 ^b^	40.43 ± 1.33 ^b^	42.45 ± 0.73 ^b^	42.98 ± 0.62 ^b^	45.44 ± 0.81 ^a^
	Mix	37.23 ± 2.12 ^b^	39.11 ± 1.03 ^b^	39.98 ± 0.75 ^b^	45.57 ± 0.78 ^a^	44.12 ± 1.51 ^a^
	1MIC Car	33.73 ± 1.12 ^b^	36.86 ± 1.69 ^ab^	40.41 ± 1.38 ^a^	40.87 ± 0.82 ^a^	42.46 ± 1.15 ^a^
	2MIC Car	40.66 ± 1.17 ^b^	41.96 ± 0.95 ^b^	40.31 ± 2.12 ^b^	49.07 ± 0.76 ^a^	48.50 ± 1.73 ^a^
	1MIC OCG*_n_*-2	45.09 ± 0.83 ^b^	43.21 ± 0.98 ^b^	44.16 ± 2.31 ^b^	37.08 ± 2.27 ^c^	50.23 ± 1.93 ^a^
	2MIC OCG*_n_*-2	51.20 ± 1.12 ^a^	44.89 ± 1.83 ^b^	48.97 ± 0.95 ^a^	49.05 ± 1.83 ^a^	48.45 ± 0.77 ^a^
a*	C	11.90 ± 0.61 ^a^	10.47 ± 0.77 ^b^	9.01 ± 0.22 ^c^	4.25 ± 0.34 ^d^	3.21 ± 0.37 ^e^
	OVA	10.81 ± 0.38 ^a^	7.26 ± 0.37 ^c^	9.14 ± 0.16 ^b^	6.04 ± 0.33 ^d^	4.15 ± 0.12 ^e^
	Mix	11.24 ± 0.38 ^a^	9.73 ± 0.22 ^b^	7.35 ± 0.28 ^c^	5.85 ± 0.87 ^d^	3.29 ± 0.11 ^e^
	1MIC Car	11.75 ± 0.46 ^a^	10.64 ± 0.25 ^b^	7.23 ± 0.60 ^c^	5.73 ± 0.38 ^d^	3.77 ± 0.16 ^e^
	2MIC Car	10.83 ± 0.13 ^a^	9.46 ± 0.76 ^b^	8.96 ± 0.39 ^b^	7.18 ± 0.19 ^c^	4.06 ± 0.15 ^d^
	1MIC OCG*_n_*-2	14.23 ± 0.33 ^a^	13.44 ± 0.44 ^a^	9.66 ± 0.17 ^b^	5.84 ± 0.17 ^c^	3.24 ± 0.11 ^d^
	2MIC OCG*_n_*-2	15.23 ± 0.41 ^a^	14.89 ± 0.61 ^a^	14.05 ± 0.44 ^a^	9.38 ± 0.28 ^b^	8.91 ± 0.12 ^b^
b*	C	7.67 ± 0.46 ^c^	7.24 ± 0.51 ^c^	7.79 ± 0.11 ^c^	9.12 ± 0.49 ^b^	11.72 ± 0.72 ^a^
	OVA	8.88 ± 0.52 ^b^	8.83 ± 0.32 ^b^	9.60 ± 0.37 ^b^	10.70 ± 0.74 ^ab^	11.80 ± 0.49 ^a^
	Mix	9.12 ± 0.42 ^b^	9.89 ± 0.42 ^b^	10.27 ± 0.49 ^b^	11.29 ± 0.53 ^ab^	12.10 ± 0.22 ^a^
	1MIC Car	9.46 ± 0.67 ^b^	9.72 ± 0.14 ^b^	10.02 ± 0.23 ^b^	11.70 ± 0.75 ^a^	12.60 ± 0.77 ^a^
	2MIC Car	8.23 ± 0.45 ^b^	8.41 ± 0.28 ^b^	8.39 ± 0.18 ^b^	9.84 ± 0.36 ^a^	10.43 ± 0.41 ^a^
	1MIC OCG*_n_*-2	12.38 ± 0.49 ^b^	13.30 ± 0.57 ^b^	13.53 ± 0.63 ^b^	14.2 ± 0.82 ^ab^	15.44 ± 0.43 ^a^
	2MIC OCG*_n_*-2	8.02 ± 0.13 ^c^	8.93 ± 0.31 ^b^	8.84 ± 0.18 ^b^	9.07 ± 0.31 ^b^	10.22 ± 0.27 ^a^

**Table 2:** The formulation and final effective concentration of different coating solutions.

Treatment	Abbreviation	Nanoparticle (mg/mL)	OVA (mg/mL)	Car (mg/mL)
untreated samples	C	0	0	0
samples coated with OVA	OVA	0	0.24	0
samples coated with a physical mixture of OVA and Car	Mix	0	0.24	0.16
samples coated with 1 MIC free Car solution	1MIC Car	0	0	0.39
samples coated with 2 MIC free Car solution	2MIC Car	0	0	0.78
samples coated with 1 MIC OCG*_n_*-2 solution	1MIC OCG*_n_*-2	0.40	0.24	0.16
samples coated with 2 MIC OCG*_n_*-2 solution	2MIC OCG*_n_*-2	0.80	0.48	0.32

## Data Availability

The data presented in this study are openly available in article.
